# Endotoxin Activity Assay-Guided Patient Selection for Polymyxin B Hemoperfusion: Lessons from the TIGRIS Trial and Future Directions

**DOI:** 10.3390/healthcare13202603

**Published:** 2025-10-16

**Authors:** Toshiaki Iba, Hideshi Okada, Takahiro Miki, Michio Mineshima, Isao Nagaoka

**Affiliations:** 1 Faculty of Medical Science, Juntendo University, Urayasu 279-0013, Japan; m.mineshima.pb@juntendo.ac.jp (M.M.); nagaokai@juntendo.ac.jp (I.N.); 2 Department of Emergency and Disaster Medicine, Gifu University Graduate School of Medicine, Gifu 501-1113, Japan; okada.hideshi.a4@f.gifu-u.ac.jp; 3 Department of Clinical Engineer, Nihon University School of Medicine, Tokyo 173-8610, Japan; miki.takahiro@nihon-u.ac.jp

**Keywords:** polymyxin B hemoperfusion, endotoxin, sepsis, shock, endotoxin activity assay

## Abstract

Sepsis and septic shock remain leading global causes of mortality, with endotoxin from Gram-negative bacteria playing a central role in their pathophysiology. Polymyxin B hemoperfusion (PMX-HP) was developed as an adjunctive therapy to directly remove circulating endotoxin in patients with sepsis and septic shock. Early clinical trials yielded conflicting results, largely due to challenges in patient selection. The endotoxin activity assay (EAA) has been investigated as a biomarker to identify patients most likely to benefit, but its limitations include indirect measurement, variability, and poor specificity. The recently completed TIGRIS trial, which enrolled septic shock patients with intermediate EAA values (0.60–0.89) and high organ dysfunction, demonstrated a significant survival benefit, thereby validating a targeted, precision medicine approach. This review critically appraises the role of EAA in guiding PMX-HP, highlights the lessons learned from the TIGRIS trial, and discusses complementary strategies such as integrating additional biomarkers, organ dysfunction scoring, and clinical phenotyping. Future research should embed EAA within multi-dimensional frameworks to optimize patient selection and establish PMX-HP as a precision therapy for endotoxemic sepsis and septic shock.

## 1. Introduction

Septic shock is a leading cause of mortality worldwide, characterized by profound inflammation, circulatory collapse, and multiple organ failure. Endotoxin (lipopolysaccharide, LPS) derived from Gram-negative bacteria is a central mediator of sepsis pathophysiology, driving immune dysregulation and vascular injury.

For over 30 years, polymyxin B hemoperfusion (PMX-HP) has been studied in numerous clinical trials. While EUPHAS (2009) [1] suggested a survival benefit, ABDO-MIX (2015) [2] and EUPHRATES (2018) [3] did not confirm mortality reduction, highlighting challenges in patient selection. Post hoc analyses of EUPHRATES suggested that patients with intermediate EAA values (0.60–0.89) may derive the most benefit, laying the foundation for the TIGRIS trial, which confirmed improved outcomes in this subgroup [4]. The question remains: Is EAA an appropriate tool to guide PMX-HP?

## 2. EAA: Principle and Clinical Use

**Figure 1:**
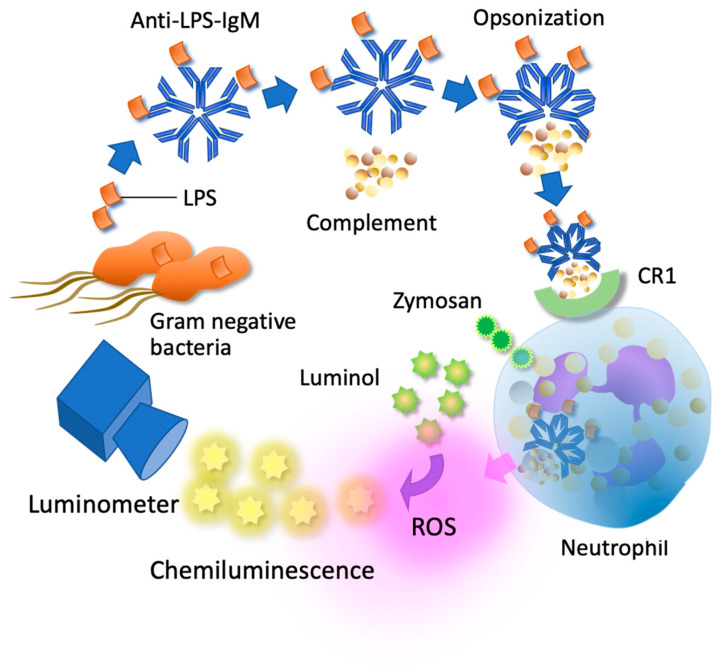
Principle of endotoxin activity assay. Endotoxin (LPS) from Gram-negative bacteria binds to anti-LPS monoclonal antibodies, forming immune complexes. Complement subsequently attaches to these complexes, leading to opsonization. Neutrophils recognize and phagocytose the opsonized immune complexes, which trigger the production of reactive oxygen species (ROS). The generated ROS reacts with luminol contained in the assay reagent, producing chemiluminescence that can be detected using a luminometer. In addition, zymosan in the reagent is phagocytosed by neutrophils and further enhances ROS production. LPS: lipopolysaccharide, CR1: complement receptor 1.

The selection of appropriate patients is mandatory for success in clinical trials [5]. The EAA is a chemiluminescence-based test that measures the neutrophil oxidative burst upon stimulation with anti-endotoxin antibody complexes [6] (Figure 1). Results are expressed as a unitless value ranging from 0.00 to 1.00, with values below 0.40 indicating a low probability of endotoxemia, values between 0.40 and 0.59 representing an indeterminate zone, and values of 0.60 or higher suggesting the presence of endotoxemia [7]. In clinical research, an EAA value ≥ 0.60 has frequently been used as the threshold for eligibility for PMX-HP, based on its presumed correlation with circulating endotoxin burden. Despite this rationale, the assay has important limitations. It provides only an indirect measurement, reflecting neutrophil responsiveness rather than absolute endotoxin concentration. The results can be influenced by confounding factors such as neutrophil dysfunction, immune paralysis, or prior treatment interventions. Moreover, elevated EAA levels are not specific to Gram-negative infections, as high values may also occur in Gram-positive sepsis or sterile inflammatory conditions. In addition, the dynamic variability of endotoxin release means that a single measurement may misclassify patients, thereby limiting its reliability as a stand-alone biomarker.

The role of EAA in clinical studies has been explored in several pivotal trials. The EUPHAS trial, conducted in patients with abdominal sepsis, reported improved hemodynamics and survival with PMX-HP, though EAA was not used for patient selection, raising concerns about generalizability. In contrast, the larger ABDO-MIX trial failed to show a mortality benefit, in part because patients were not screened with EAA and because of high rates of incomplete treatment. The EUPHRATES trial represented a major step forward by enrolling patients with septic shock and EAA ≥ 0.60, but the overall results were neutral. Importantly, a post hoc analysis revealed a potential benefit in the subgroup with multiple organ dysfunction and intermediate EAA values (0.60–0.89). Building on this finding, the TIGRIS trial adopted a biomarker-enriched approach, enrolling patients with septic shock, intermediate EAA values, and severe organ dysfunction. This strategy translated into a significant survival benefit, demonstrating that careful patient selection can convert neutral results into positive outcomes and underscoring the importance of EAA-guided precision medicine in sepsis research.

## 3. EAA and Clinical Studies

Several clinical trials have examined the impact of PMX-HP in sepsis and septic shock, with results shaped largely by differences in patient selection and methodology. The EUPHAS trial, a small Italian randomized controlled study involving 64 patients with abdominal sepsis, demonstrated improved hemodynamics and survival with PMX-HP, although patients were not selected based on EAA, raising concerns about the generalizability of its findings. The subsequent ABDO-MIX trial, a larger French multicenter study of 243 patients, failed to show a mortality benefit. The absence of EAA screening and the high proportion of incomplete treatments were major limitations that reduced the reliability of its conclusions. The EUPHRATES trial, the largest to date with 450 patients across North America, restricted enrollment to septic shock patients with EAA ≥ 0.60. Although the primary outcome of 28-day mortality was negative overall, a post hoc analysis identified a subgroup of patients with multiple organ dysfunction score (MODS) > 9 and intermediate EAA values (0.60–0.89) who appeared to derive benefit from PMX-HP. Building directly on this observation, the TIGRIS trial adopted a biomarker-enriched approach by enrolling patients with septic shock, intermediate EAA values, and severe organ dysfunction (MODS > 9 or sequential organ failure assessment (SOFA) score > 11). This trial employed a Bayesian design and, in contrast to earlier studies, demonstrated both a 28-day survival advantage and a significant reduction in 90-day mortality. With an adjusted odds ratio of 0.54 for 90-day mortality, TIGRIS provided compelling evidence that targeted patient selection can transform previously neutral findings into meaningful clinical success. This study suggested improved outcomes by applying a targeted therapy approach, with the 90-day mortality reduction (adjusted OR 0.54) being noteworthy.

## 4. Limitation of EAA

EAA has emerged as a promising biomarker for identifying septic patients with endotoxemia, yet its sufficiency as the sole determinant of eligibility for PMX-HP remains controversial. The principal strength of EAA lies in its ability to provide rapid, point-of-care information about endotoxin-related immune activation. Large diagnostic studies such as the MEDIC trial demonstrated that EAA had a strong negative predictive value for Gram-negative infection and was associated with sepsis severity and mortality [8] (Table 1). Observational work in Japan similarly showed that EAA not only identified patients with elevated endotoxin activity but also tracked decreases following PMX-HP [6]. Most convincingly, the recent TIGRIS trial demonstrated that restricting enrollment to patients with intermediate EAA values (0.60–0.89) and high organ dysfunction converted previously neutral results into a significant survival benefit [9]. These findings highlight the utility of EAA in enriching trial populations and guiding precision therapy.

Nonetheless, significant limitations constrain the use of EAA as a stand-alone biomarker. The assay provides only an indirect measure of endotoxin activity, as it depends on neutrophil oxidative responses that may be impaired in critical illness. Neutrophil dysfunction can attenuate the oxidative burst and produce spuriously low results in sepsis-induced immunoparalysis, bone marrow suppression, or metabolic disorders such as glucose-6-phosphate dehydrogenase (G6PD) deficiency. Such false negatives may obscure the presence of clinically relevant endotoxemia and lead to under-recognition of patients who might otherwise benefit from targeted therapies like PMX-HP. Conversely, elevated EAA values are not specific to Gram-negative infections, as increases may also be observed in Gram-positive sepsis or sterile inflammatory states, thereby raising concerns about false positives and limiting diagnostic specificity. Taken together, these constraints highlight that EAA should be interpreted within the broader clinical and laboratory context rather than being used as an isolated determinant of therapy [6]. False positive EAA elevations may lead to inappropriate patient selection for PMX-HP if used in isolation. Importantly, the EUPHRATES trial revealed that patients with very high EAA (≥0.90) failed to benefit from PMX-HP, suggesting assay saturation or distinct pathophysiological processes at extreme endotoxin burdens [3]. Technical variability, temporal fluctuations in endotoxin release, and limited global availability further complicate routine clinical application.

Taken together, EAA is best viewed as a valuable enrichment tool rather than a definitive biomarker [14]. To maximize the therapeutic potential of PMX-HP, future strategies should integrate EAA with complementary biomarkers, organ dysfunction scores, and clinical phenotyping, thereby embedding it within a broader precision medicine framework.

## 5. Alternative Test to EAA

While EAA has value as a bedside biomarker, its limitations highlight the need for alternative or complementary approaches to refine patient selection for PMX-HP. Direct endotoxin quantification remains the most intuitive strategy, with methods such as the Limulus Amebocyte Lysate (LAL) assay [15] traditionally employed to measure circulating lipopolysaccharide. However, LAL is susceptible to plasma inhibitors and often underestimates biologically active endotoxin. Newer technologies, including mass spectrometry–based lipid A detection, offer higher specificity and may overcome prior limitations [16]. Beyond direct endotoxin measurement, host-response biomarkers provide critical insight into sepsis pathophysiology. Elevated cytokines such as IL-6 and TNF-α reflect systemic hyperinflammation, while presepsin and procalcitonin serve as indicators of bacterial burden and immune activation [12,17]. Combining EAA with such biomarkers may yield improved accuracy in identifying patients with both significant endotoxemia and heightened susceptibility to organ injury.

Genomic and transcriptomic profiling is another promising avenue. Recent studies have identified distinct sepsis endotypes, ranging from hyperinflammatory to immunosuppressed phenotypes, that predict differential outcomes and therapeutic responses [18]. Integrating EAA within such molecular frameworks could ensure that PMX-HP is deployed in biologically plausible contexts. Clinical phenotyping remains equally critical: patients with refractory septic shock, high vasopressor requirements, and intra-abdominal Gram-negative infections may represent ideal candidates irrespective of EAA level, aligning with pragmatic bedside decision-making [19]. Composite risk models that combine EAA values with clinical severity indices such as sequential organ failure assessment (SOFA) or MODS scores may offer a balanced approach, as demonstrated by the enriched population strategy in the TIGRIS trial.

Recent advances in genomics and transcriptomics have begun to identify novel biomarkers that could refine patient stratification for PMX-HP. For example, miR-146a has been shown to negatively regulate Toll-like receptor (TLR)-4/nuclear factor (NF)-κB signaling, with increased expression attenuating cardiac dysfunction and systemic inflammation in murine models of polymicrobial sepsis through suppression of IRAK1 (Interleukin-1 receptor-associated kinase) and TRAF6 (TNF receptor-associated factor 6) [20]. In human studies, elevated serum miR-146a correlates with more severe disease status and poorer prognosis in sepsis, especially when complicated by acute lung injury. Similarly, miR-150 (MicroRNA 150) levels are reduced in patients with sepsis, correlating negatively with markers of organ dysfunction (e.g., renal impairment) and 28-day mortality; mechanistically, miR-150 targets NF-κB and suppresses LPS-induced endothelial cell inflammation and apoptosis [21]. Meta-analyses of sepsis biomarker studies also include miR-146a, miR-150 among panels (alongside miR-21, miR-223, etc.) with moderate to high diagnostic or prognostic value [22].

Regarding genetic polymorphisms, some evidence pertains to TLR4 variants (Asp299Gly, Thr399Ile) in sepsis. A recent meta-analysis suggests that Asp299Gly may have a marginal protective effect under certain genetic models, though findings are inconsistent and not definitively significant [23]. Another study found that protein expression levels of TLR4 and its adaptor TIRAP (TIR domain containing adaptor protein) correlated strongly with sepsis severity, suggesting that expression assays may be more clinically relevant than genotyping alone in some contexts [24]. However, there is no clear evidence linking them yet to endotoxin burden or PMX-HP responsiveness. Therefore, they should be presented as hypothetical or emerging candidates rather than established biomarkers.

Taken together, these alternatives illustrate that reliance on a single biomarker is insufficient. Future strategies should adopt a multi-dimensional precision medicine framework, integrating EAA with biochemical, genomic, and clinical data to optimize patient selection and therapeutic efficacy.

## 6. Lessons from the TIGRIS Trial

The TIGRIS trial represents a pivotal milestone in the long and controversial history of PMX-HP [25]. Unlike earlier studies that produced conflicting outcomes, TIGRIS adopted a biomarker-enriched, precision medicine strategy by restricting enrollment to septic shock patients with intermediate endotoxin activity (EAA 0.60–0.89) and high organ dysfunction (MODS > 9 or SOFA > 11). This approach translated into meaningful clinical benefit, with a reported 90-day absolute risk reduction of 17.4% and a number needed to treat of 8.1. These findings reinforce the concept that careful patient selection can convert negative trials into positive results, echoing the post hoc analyses of EUPHRATES, which first identified this responsive subgroup [3]. However, TIGRIS also highlights important limitations: the benefit was confined to a narrow patient subset, the assay itself is not universally available, and the Bayesian trial design, while efficient, may complicate regulatory acceptance. Collectively, TIGRIS underscores both the potential and challenges of biomarker-guided sepsis therapy.

## 7. Alternative Patient Selection

While the EAA has provided a framework for targeted patient selection in PMX-HP trials, its limitations and limited availability underscore the need for complementary or alternative approaches. The results of the TIGRIS trial demonstrated that mortality in septic shock is closely linked to the severity and pattern of organ dysfunction, independent of EAA measurement [9]. Patients with high SOFA scores (>11) or MODS scores (>9) exhibited particularly poor prognoses, with mortality rates ranging from 48% to 75%. These findings suggest that organ dysfunction severity alone can serve as a pragmatic criterion for identifying candidates most likely to benefit from PMX-HP.

Specific organ dysfunctions appear to have prognostic significance. Acute kidney injury (KDIGO stage 2–3), hepatic dysfunction (bilirubin ≥ 2 mg/dL), respiratory failure requiring mechanical ventilation, and coagulation abnormalities (platelet count <100,000/µL) were strongly associated with elevated endotoxin severity scores and high mortality [26]. These features overlap with the δ phenotype of sepsis, a subgroup defined by profound multiorgan failure and the worst outcomes [26]. Importantly, such clinical phenotypes can guide therapy in settings where EAA is not available, aligning PMX-HP deployment with patient subgroups most likely to reflect endotoxin-mediated pathology.

Real-world evidence further supports the use of clinical severity scores as a selection tool. Retrospective analyses from Japan found that PMX-HP was associated with improved survival in patients with SOFA scores ≥ 7, with the strongest signal in those with scores between 10 and 12 [27,28]. These results align with post hoc analyses of the EUPHRATES trial, which similarly suggested benefit in patients with high organ dysfunction burden despite intermediate endotoxin activity [3,4]. Molinari and colleagues [29] subsequently proposed that in the absence of EAA, PMX-HP should be considered for patients with septic shock characterized by high SOFA or MODS scores and distinctive patterns of renal, hepatic, respiratory, and hematologic dysfunction.

Taken together, these findings highlight that while EAA can enrich trial populations, severity-based patient selection using organ dysfunction scores and clinical phenotyping represents a viable alternative strategy. This approach not only reflects the pathophysiological burden of endotoxemia but also provides a practical framework for implementing PMX-HP in diverse healthcare systems where biomarker testing may not be accessible. By integrating lessons from TIGRIS with real-world evidence and sepsis phenotyping, patient selection strategies can move toward a more pragmatic yet precise model, thereby strengthening the translational potential of PMX-HP in routine clinical practice.

## 8. Future Perspectives

Future perspectives on optimizing PMX-HP therapy in sepsis center on advancing beyond single-biomarker approaches and validating findings like those of the TIGRIS trial in broader, more diverse patient populations. While EAA-guided enrichment markedly improved trial outcomes, reliance on a single, functionally indirect assay is limiting. Upcoming research should incorporate multi-biomarker panels—combining EAA with host-response indicators (e.g., presepsin, procalcitonin, IL-6) and emerging genomic and transcriptomic endotyping—to better capture both burden and biological context of endotoxemia and immune dysfunction. Adaptive clinical trial designs, especially Bayesian enrichment strategies, can maximize efficiency and personalization. Implementation studies must evaluate the feasibility, cost-effectiveness, and real-world logistics of incorporating EAA and related assays, particularly in resource-limited settings. Regulatory acceptance may require harmonized protocols and demonstration of survival benefit in pragmatic trials. Ultimately, integrating clinical phenotyping with molecular and functional biomarkers will be essential to establish PMX-HP as a robust, precision sepsis intervention and to guide global adoption. As PMX-HP moves toward precision medicine, collaborative international efforts and harmonized trial frameworks will set a new standard for sepsis care, aligning biomarker discovery, patient selection, and therapeutic deployment for maximal patient benefit [30].

## 9. Conclusions

The evolving evidence base demonstrates that while the Endotoxin Activity Assay (EAA) has enabled more precise patient selection and generated positive randomized data in the TIGRIS trial, its limitations as a stand-alone tool remain clear. EAA should be regarded as an enrichment tool rather than a stand-alone biomarker. Successful, widespread adoption of PMX-HP for septic shock will require integrating EAA with clinical severity scores, other biomarkers, and possibly genomic data to guide therapy. Ultimately, a multi-dimensional, precision medicine approach—validated in diverse patient populations—will be essential to maximizing clinical benefit, ensuring cost-effectiveness, and achieving regulatory acceptance worldwide. This integrated strategy offers hope to improve outcomes for patients with endotoxemic septic shock.

## Figures and Tables

**Table 1:** Summary of Endotoxin Activity Assay Study.

Study/Trial	Population	EAA Threshold	Key Findings	Reference
**MEDIC Trial (2004)**	ICU patients with SIRS, *n* = 857	≥0.40 (intermediate), ≥0.60 (high)	EAA correlated with Gram-negative infection risk, severity, and mortality; NPV for Gram-negative infection was 98.6%.	[8]
**EAA-J Trial (2007)**	ICU patients with sepsis, *n* = 215	≥0.60	Distribution: 22% <0.40, 35% 0.40–0.59, 43% ≥0.60. PMX-HP reduced EAA by ~26%.	[6]
**Novelli et al. (2010)**	Post-surgical sepsis, *n* = 17	>0.60 for PMX-DHP treatment	EAA-guided PMX therapy reduced EAA levels; hemodynamic improvement was observed.	[10]
**Yaguchi et al.** **(2012)**	Severe sepsis, *n* = 210	≥0.40 (intermediate), ≥0.60 (high)	Positive cultures for GNs: 0.47 (IQR 0.27) vs. negative: 0.34 (IQR 0.22); *p* < 0.0001	[11]
**Ikeda et al.** **(2014)**	ICU patients *n* = 314	≥0.40 (intermediate), ≥0.60 (high)	EAA levels were 0.39 ± 0.25 (mean ± SD) in patients, 0.52 ± 0.22 in sepsis, and 0.10 ± 0.09 in healthy volunteers	[12]
**Bottiroli et al.** **(2017)**	Septic shock *n* = 107	≥0.40 (intermediate), ≥0.60 (high)	Positive cultures for GNs: 0.63 ± 0.18 (mean ± SD) vs. negative: 0.53 ± 0.22; *p* < 0.05	[13]
**EUPHRATES Trial (2018)**	Septic shock with EAA ≥ 0.60, *n* = 450	≥0.60	No overall mortality benefit. Post hoc: benefit in MODS >9 and EAA 0.60–0.89 subgroup.	[3]
**TIGRIS Trial (2025)**	Septic shock, EAA 0.60–0.89, MODS > 9, *n* = 151	0.60–0.89	28-day ARR 6.4%; 90-day ARR 17.4%; NNT = 8.1. Confirmed benefit in biomarker-defined subgroup.	[9]

ICU: intensive care unit, SIRS: systemic inflammatory response syndrome, EAA: endotoxin activity assay, NPV: negative predictive value, PMX-HP: Polymyxin B hemoperfusion, MODS: multiple organ dysfunction syndrome, IQR: inter-quartile range, SD: standard deviation.

## Data Availability

No new data were created or analyzed in this study.
